# Thoracopagus Conjoined Twins: A Case Report

**DOI:** 10.5402/2011/238360

**Published:** 2010-11-28

**Authors:** Mehmet A. Osmanağaoğlu, Turhan Aran, Süleyman Güven, Cavit Kart, Özgür Özdemir, Hasan Bozkaya

**Affiliations:** Department of Obstetrics and Gynecology, Karadeniz Technical University, Trabzon 61080, Turkey

## Abstract

*Objective*. Conjoined twin is a rarely seen congenital anomaly together with severe mortality and morbidity. The more common types of conjoined twins include the thoracopagus type, where the fusion is anterior, at the chest, and involves the heart. We are reporting one case of conjoined thoracopagus twins diagnosed by ultrasonography at 11 weeks. *Case Report*. In a multigravid pregnant woman who has been admitted to our clinic with a diagnosis of conjoined twins, thoracopagus, by ultrasonography at an 11-week gestation, termination of the pregnancy was performed. *Conclusion*. Making an early diagnosis with ultrasonographic examination gives the parents a chance to elect pregnancy termination.

## 1. Introduction

Conjoined twins represent one of the rarest forms of twin gestation. They occur in roughly 1 in every 200 identical twin pregnancies and are always identical. The incidence ranges from 1 in 50 000 to 1 in 100 000 live births [1]. Because this situation carries high risk, early diagnosis and management of delivery is extremely important. The role of ultrasound in early diagnosis and management are discussed.

## 2. Case Report

**Figure 1:**
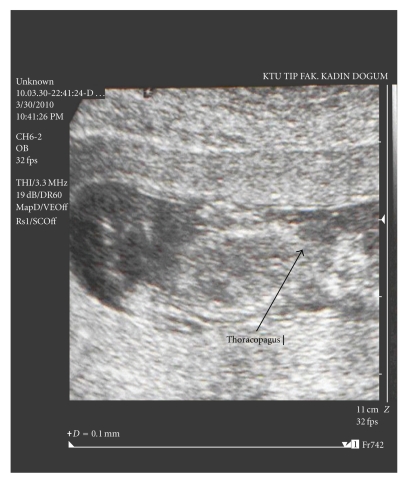
Image of conjoined twins (thoracopagus) at 11 weeks' gestation.

**Figure 2:**
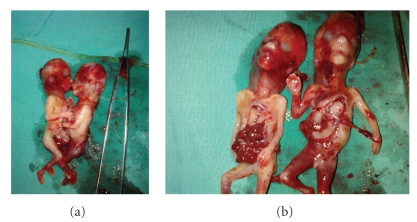
Image of conjoined twins after vaginal delivery at 11 weeks' gestation.

A 31-year-old multigravid woman was referred to our university hospital at an 11-week gestation because of a conjoined twin (thoracopagus) diagnosed by ultrasonography. Her last menstrual date was unknown. She had no personal or family history of twins. Sonography was performed and two fetuses with 2 arms, 2 legs, and 2 heads were visualized. The twins were joined at the thorax and upper abdomen. There was a single umbilical cord, and only one fetal heart was observed (Figure 1). The placenta was localized anteriorly, and one artery and one vein were seen in the umbilical cord. On the basis of these findings, the diagnosis of terata anacatadidyma, thoracopagus, conjoined twins was made (Figures 2(a) and 2(b)), and the parents were informed about the malformation and the twins' poor chance for survival. The parents decided to terminate the pregnancy. A written informed consent was taken from the family, and the termination of pregnancy was approved by the Medical Ethics Committee. The next two days, after induction of labor with prostaglandin, a vaginal delivery of the conjoined twins was achieved without complication.

## 3. Discussion

Conjoined twins are classified according to the most prominent site of conjunction: thorax (thoracopagus), abdomen (omphalopagus), sacrum (pygopagus), pelvis (ischiopagus), skull (cephalopagus), and back (rachipagus). Depending on the aspect of the embryonic disc, the most common types are thoracopagus (19%) [3] (Table 1). Its etiology is unknown, but an incomplete division of the zygote between 13th and 15th days after fertilization probably occurs [4]. The overall survival rate for conjoined twins is approximately 25% [5]. The condition is more frequently found among females, with a ratio of 3 : 1 [4]. Two theories have been proposed to explain this observation: the process of X-inactivation overlaps with the timing of monozygotic twinning and thus may directly contribute to development of monozygotic twins, and the XX karyotype may confer a survival benefit [2].

Two contradicting theories exist to explain the origins of conjoined twins. The traditional theory is fission, in which the fertilized egg splits partially and conjoined twins represent delayed separation of the embryonic mass after day 12 of fertilization. The second theory is fusion, in which a fertilized egg completely separates, but stem cells (which search for similar cells) find like-stem cells on the other twin and fuse the twins together [4, 6, 7]. Conjoined twins share a single common chorion, placenta, and amniotic sac, although these characteristics are not exclusive to conjoined twins as there are some monozygotic but nonconjoined twins that also share these structures in utero [4, 6].

Early diagnosis of conjoined twins was previously reported, but not before the 10th week of gestation [8]. On careful transvaginal sonography and serial scanning, there appears to be an inability to separate between the anatomical parts of the fetuses. Once conjoined twins have been diagnosed, characterization of the type and severity of the abnormality can be performed with ultrasound, three-dimensional ultrasound, computed tomography, or magnetic resonance imaging [9, 10]. Termination of pregnancy can be offered to the family. In the present study, the diagnosis has been performed in the first trimester, and because the family has chosen termination of this pregnancy, further diagnostic intervention has not been considered. Surgery to separate conjoined twins may range from relatively simple to extremely complex, depending on the point of attachment and the internal parts that are shared. Most cases of separation are extremely risky and life-threatening.

In conclusion, conjoined twins are associated with a high perinatal mortality; therefore, making an early diagnosis with ultrasonographic examination of conjoined twins gives the parents a chance to elect pregnancy termination.

## Figures and Tables

**Table 1:** Embryologic classification of conjoined twins [2].

Embryonic aspect	Type	Incidence	Primordium	Extent of union	Separability
Ventral (87%)	—	—	—	—	—

Rostral (48%)	Cephalopagus	11%	Oropharyngeal membrane	Top of head to umbilicus	None
	Thoracopagus	19%	Heart	Thorax, upper abdomen, conjoined heart	Rare
	Omphalopagus	18%	Diaphragm	Thorax, upper abdomen, separate hearts	Likely 82% success

Caudal (11%)	Ischiopagus	11%	Cloacal membrane	Lower abdomen, genitourinary tract	Likely 63% success

Lateral	Parapagus	28%	Cloacal membrane (2 notochords?)	Pelvis, variable trunk, diprosopus 2 faces, dicephalus 2 heads	Rare

Dorsal (13%)	Craniopagus	5%	Cranial neuropore	Cranial vault	Unlikely without sequelae
	Rachipagus	2%	Neural tube (mid-portion)	Vertebral column	None reported
	Pygopagus	6%	Caudal neuropore	Sacrum	Likely 68% success
